# Serine and glycine metabolism-related gene expression signature stratifies immune profiles of brain gliomas, and predicts prognosis and responses to immunotherapy

**DOI:** 10.3389/fphar.2022.1072253

**Published:** 2022-11-17

**Authors:** Siliang Chen, Shuxin Zhang, Wentao Feng, Junhong Li, Yunbo Yuan, Wenhao Li, Zhihao Wang, Yuan Yang, Yanhui Liu

**Affiliations:** ^1^ Department of Neurosurgery, West China Hospital of Sichuan University, Chengdu, China; ^2^ Department of Head and Neck Surgery, Sichuan Cancer Hospital and Institute, Sichuan Cancer Hospital, School of Medicine, University of Electronic Science and Technology of China, Chengdu, China; ^3^ Department of Neurosurgery, Chengdu Second People’s Hospital, Chengdu, China

**Keywords:** serine, glycine, metabolism, glioma, prognosis, immune infiltration, tumor microenvironment, immune checkpoint inhibitor

## Abstract

Glioma is one of the most lethal cancers and causes more than 200,000 deaths every year. Immunotherapy was an inspiring therapy for multiple cancers but failed in glioma treatment. The importance of serine and glycine and their metabolism has been well-recognized in the physiology of immune cells and microenvironment in multiple cancers. However, their correlation with prognosis, immune cells, and immune microenvironment of glioma remains unclear. In this study, we investigated the relationships between the expression pattern of serine and glycine metabolism-related genes (SGMGs) and clinicopathological features, prognosis, and tumor microenvironment in glioma based on comprehensive analyses of multiple public datasets and our cohort. According to the expression of SGMGs, we conducted the consensus clustering analysis to stratify all patients into four clusters with remarkably distinctive clinicopathological features, prognosis, immune cell infiltration, and immune microenvironment. Subsequently, a serine and glycine metabolism-related genes signature (SGMRS) was constructed based on five critical SGMGs in glioma to stratify patients into SGMRS high- and low-risk groups and tested for its prognostic value. Higher SGMRS expressed genes associated with the synthesis of serine and glycine at higher levels and manifested poorer prognosis. Besides, we confirmed that SGMRS was an independent prognostic factor and constructed nomograms with satisfactory prognosis prediction performance based on SGMRS and other factors. Analyzing the relationship between SGMRS and immune landscape, we found that higher SGMRS correlated with ‘hotter’ immunological phenotype and more immune cell infiltration. Furthermore, the expression levels of multiple immunotherapy-related targets, including PD-1, PD-L1, and B7-H3, were positively correlated with SGMRS, which was validated by the better predicted response to immune checkpoint inhibitors. In conclusion, our study explored the relationships between the expression pattern of SGMGs and tumor features and created novel models to predict the prognosis of glioma patients. The correlation of SGMRS with immune cells and microenvironment in gliomas suggested an essential role of serine and glycine metabolism in reforming immune cells and microenvironment. Finally, the results of our study endorsed the potential application of SGMRS to guide the selection of immunotherapy for gliomas.

## Introduction

Glioma is one of the most life-threatening tumors and accounts for approximately 80% of malignant tumors in the central nervous system (Ostrom et al., 2021). The prognosis of glioma patients remains poor even after a complete standard treatment regime consisting of surgery, chemotherapy, and radiotherapy (Stupp et al., 2005). For example, the median overall survival of patients with glioblastoma, which is the most aggressive glioma and accounts for nearly 50% of all gliomas, is fewer than two years after thorough treatment (Chinot et al., 2014; Gilbert et al., 2014; Stupp et al., 2015; Omuro et al., 2022). Therefore, exploring novel therapy to improve the prognosis of glioma patients is urgently needed and attracts abundant researchers to devote themselves to it. In recent years, the applications of immunotherapy, which aims to enhance anti-tumor immunity delivered by immune cells, have been endorsed by lots of studies in multiple cancers, including melanoma (Larkin et al., 2015), non-small-cell lung cancer (Reck et al., 2016), gastric cancer (Janjigian et al., 2021), and renal cell carcinoma (Choueiri et al., 2021a). Multiple randomized clinical trials were also devoted to evaluating the efficacy of immune checkpoint inhibitors (ICIs) in the treatment of glioblastoma, but all these attempts eventually failed (Reardon et al., 2020; Lim et al., 2022; Omuro et al., 2022). The immunologically quiescent environment of the brain is considered an important reason for these failures. The blood-brain barrier not only prevents the majority of antitumor drugs out of brain, but also blocks most peripheral immune cells from entering central nervous system. Besides, regulatory T (Treg) cells in tumor microenvironment of glioma functions to deliver immunosuppressive effects by exhausting cytotoxic T cells, which is another reason for the failure of immunotherapy to activate T cells (Colombo and Piconese, 2007). However, metastatic brain tumors located in similar environments with gliomas can benefit from ICIs therapy (Tawbi et al., 2018; Hendriks et al., 2019), indicating that the unique immune microenvironment of gliomas may result in resistance to ICIs. Adjuvant ICIs for glioblastoma would reshape the immune microenvironment and enhance anti-tumor immunity (Cloughesy et al., 2019; Schalper et al., 2019). Therefore, investigating potential pathways that influence the immune microenvironment of gliomas can provide novel methods to reshape the immune landscape and subsequently enhance anti-tumor immunity, reinforce the efficacy of immunotherapy, and improve prognosis.

Serine and glycine, two non-essential amino acids, play critical roles in multiple cell physiological processes (Sullivan and Vander Heiden, 2017). Cells require serine and glycine *via* intracellular synthesis and uptake from the extracellular environment. The synthesis process of serine and glycine consists of two steps: *de novo* synthesis of serine branched from glycolysis and reversible interconversion from serine to glycine (Geeraerts et al., 2021), indicating the tight relationship between the metabolism processes of these two amino acids. The function of serine, glycine, and their metabolism in cancers attracted significant attention in recent years. Upregulated synthesis of serine and glycine has been demonstrated in multiple cancers, including lung cancer and glioma (Kim et al., 2015; Liao et al., 2019b). The important physiological roles of serine and glycine synthesis in tumors, including fueling nucleotide biosynthesis (Fan et al., 2019), regulating lipid metabolism (Gao et al., 2018), altering sphingolipid diversity (Muthusamy et al., 2020), and maintaining cellular redox homeostasis (Ye et al., 2014), were potential causes that drive the tumors to upregulate the synthesis of serine and glycine to meet the aberrant demand. The process of serine and glycine synthesis can generate abundant one-carbon units and replenish carbon sources for one-carbon metabolism in cancer cells (Locasale, 2013; Newman and Maddocks, 2017; Fan et al., 2019). Besides, serine and glycine are critical for the survival and growth of cancer cells (DeBerardinis, 2011; DeBerardinis and Chandel, 2016). Downregulation of serine and glycine synthesis has been shown to inhibit cancer cell proliferation (Mullarky et al., 2016; Pacold et al., 2016). Cancer cells can not only upregulate serine and glycine synthesis, but also secret extra serine and glycine to extracellular spaces to reshape tumor microenvironment (Geeraerts et al., 2021). In glioma, glycine concentration was determined as a biomarker of aggressiveness (Tiwari et al., 2020). Serine and glycine in tumor microenvironment enhanced nucleotide production and cell proliferation in brain metastasis (Ngo et al., 2020). Furthermore, serine and glycine synthesized and secreted by cancer cells play multiple roles in tumor immune microenvironment. Serine in extracellular environments inhibits the functions of macrophages and neutrophils (He et al., 2019). A high level of phosphoglycerate dehydrogenase (PHGDH), an essential enzyme for serine and glycine synthesis, can induce macrophages to immunosuppressive M2-like macrophages (Wilson et al., 2020). Serine and glycine synthesis can also switch the phenotype of macrophages to express immunosuppressive programmed death-ligand (PD-L1) by inducing the production of IL-1β (Su et al., 2018; Rodriguez et al., 2019). These findings suggest that the metabolism of serine and glycine is involved in tumorigenesis and related to the aggressiveness and immune microenvironment of cancers. However, the role of serine and glycine metabolism in malignant features and the immune landscape of glioma remains unclear and need to be further elucidated.

In this study, we comprehensively analyzed the RNA-sequence data from multiple glioma patient cohorts, including TCGA, CGGA325, CGGA693, and our institution, to investigate the relationship between serine and glycine metabolism-related genes (SGMGs) expression and clinicopathological characteristics of glioma. Moreover, we constructed a serine and glycine metabolism-related gene risk signature (SGMRS) to evaluate the clinical significance of SGMGs expression profile. Additionally, we also conducted several analyses to investigate the correlation between the expression of SGMGs and the tumor immune microenvironment landscape of glioma.

## Materials and methods

### Data sources

Gene expression profile (fragments per kilobase million, FPKM) and clinicopathological features in this study were obtained from three public databases and an own cohort. Those patients with primary oligodendroglioma, astrocytoma, and glioblastoma were included in this study. Those patients with recurrent gliomas or age <18 were exclude from this study, because these tumors occupied minority of the data set with distinctive biological features (Louis et al., 2021). The three cohorts of public databases were from the Cancer Genome Atlas (TCGA, https://portal.gdc.cancer.gov/) and the Chinese Glioma Genome Atlas (CGGA, http://www.cgga.org.cn/). The TCGA cohort contained 662 primary glioma samples, and 655 of which had complete survival data. There are two cohorts from the CGGA database, CGGA325 and CGGA 693 cohorts. The CGGA325 cohort contained 226 adult primary gliomas, and the CGGA693 cohort contained 415 primary gliomas. FPKM data of these two cohorts were downloaded from the CGGA website.

Our own cohort consisted of 77 primary glioma patients from West China Hospital (WCH). The tumor samples were obtained during tumor resection surgery and subsequently sequenced for mRNA. After that, the mRNA sequencing data was quantified and normalized to FPKM by STAR. Prognosis information of these 77 patients was obtained through regular follow-up and telephone interview. The overall survival (OS) was calculated as the time length from surgery to death or last follow-up (censored value). In preprocessing procedure, we exclude the genes with too low FPKM values (maximum FPKM <0.1 or standard deviation < 0.01, which may represent sequencing/mapping artifacts) from further analyses. Detailed clinicopathological information of these four cohorts was showed in Table 1.

**TABLE 1:** Clinicopathological characteristics of patients in TCGA, CGGA325, CGGA693, and WCH cohorts.

Characteristics	TCGA (N = 662)	CGGA325 (N = 226)	CGGA693 (N = 415)	WCH (N = 77)
Age: mean (range)	46 (18–89)	52 (22–87)	43 (19–76)	46 (19–77)
Gender				
Female	282 (42.6%)	87 (38.5%)	176 (42.4%)	30 (39.0%)
Male	380 (57.4%)	139 (61.5%)	239 (57.6%)	47 (77.0%)
NA	0	0	0	0
Histology				
Astrocytoma	341 (51.5%)	82 (36.3%)	182 (43.9%)	22 (28.6%)
Oligodendroglioma	167 (25.2%)	60 (26.6%)	94 (22.7%)	21 (27.3%)
Glioblastoma	154 (23.3%)	84 (37.2%)	139 (33.5%)	34 (44.2%)
Grade				
G2	214 (32.3%)	94 (41.6%)	134 (32.3%)	29 (37.7%)
G3	237 (35.8%)	48 (21.2%)	142 (34.2%)	14 (18.2%)
G4	154 (23.3%)	84 (37.2%)	139 (33.5%)	34 (44.2%)
NA	57 (8.6%)	0	0	0
IDH status				
Mutant	421 (63.6%)	115 (50.9%)	169 (40.7%)	42 (54.5%)
WT	236 (35.6%)	110 (48.7%)	207 (49.9%)	35 (45.5%)
NA	5 (0.8%)	1 (0.4%)	39 (9.4%)	0
1p19q Codeletion				
Codel	167 (25.2%)	54 (23.9%)	267 (64.3%)	19 (24.7%)
Non-codel	488 (73.7%)	169 (74.8%)	88 (21.2%)	43 (55.8%)
NA	7 (1.1%)	3 (1.3%)	60 (14.5%)	15 (19.5%)
TERT promoter status				
Mutant	340 (51.4%)	NA	NA	30 (39.0%)
WT	156 (23.6%)	NA	NA	23 (29.9%)
NA	166 (25.1%)	NA	NA	24 (31.2%)
MGMT promoter status				
Methylated	472 (71.3%)	97 (42.9%)	141 (34.0%)	35 (45.5%)
Unmethylated	157 (23.7%)	115 (50.9%)	195 (47.0%)	13 (16.9%)
NA	33 (5.0%)	14 (6.2%)	79 (19.0%)	29 (37.7%)
ATRX status				
Mutant	192 (29.0%)	NA	NA	22 (28.6%)
WT	459 (69.3%)	NA	NA	53 (68.8%)
NA	11 (1.7%)	NA	NA	2 (2.6%)

Abbreviation: TCGA, the cancer genome atlas; CGGA, chinese glioma genome atlas; WCH, west china hospital; IDH, isocitrate dehydrogenase; TERT, telomerase reverse transcriptase; MGMT, O6-methylguanine-DNA, methyltransferase; ATRX, alpha-thalassemia x-linked intellectual disability syndrome; WT, wild type; NA, not available.

### Consensus clustering analyses based on serine and glycine metabolism-related genes

The serine and glycine metabolism-related genes (SGMGs) were identified based on the serine and glycine metabolism pathway from PathBank (https://pathbank.org/, pathway No. SMP0000004), which contained 24 SGMGs. After excluding the genes with low expression levels, 21 SGMGs were eventually enrolled in the following analyses. The list of these 24 SGMGs was downloaded from the PubChem website (https://pubchem.ncbi.nlm.nih.gov/pathway/PathBank:SMP0000004/), and the list of SGMGs before and after exclusion was given in Supplementary Table S1. Subsequently, unsupervised consensus clustering analyses were conducted based on expression patterns of SGMGs to represent the different serine and glycine metabolism patterns in gliomas. Consensus clustering analysis was conducted using the R package ‘ConsensusClusterPlus’. Briefly, for number of clusters (k) from 2 to 10, hierarchical clustering of k clusters was performed over 1,000 random subsets of samples based on Pearson correlation. The consensus index was calculated as the frequency for which two samples were stratified into the same cluster. The optimal k was determined when gain in area under the cumulated distribution function (CDF) curve of the consensus index converged with the increase of k, under the restriction that the sample size of each cluster should not too small to study its implications. Furthermore, we performed the t-Distributed Stochastic Neighbor Embedding (tSNE) analysis to visualize the different expression patterns of SGMGs in each cluster. Besides, a naïve Bayes classifier was constructed based on the SGMGs expression and cluster labels of the TCGA cohort to classify the patients of the other three cohorts into distinctive clusters.

### Construction and validation of the serine and glycine metabolism-related genes risk signature

To elucidate the relationship between serine and glycine metabolism and glioma, we constructed a gene risk signature based on the expression of SGMGs, the serine and glycine metabolism-related genes risk signature (SGMRS). In the first step, patients of TCGA cohort were split into training and validation sets with a ratio of 6:4, and all the other three cohorts were defined as validation sets. Subsequently, we utilized the Least Absolute Shrinkage and Selection Operator (LASSO) Cox regression analysis to filter the 21 SGMGs in the training set. The SGMG was determined as critical SGMG if its coefficient was not zero at the optimal model with maximum C-indices in over 80 random repetitions of LASSO Cox regression out of 100. Moreover, we fitted a concluding multivariate Cox regression model to the training set with critical SGMGs. The serine and glycine metabolism-related genes risk signature (SGMRS) was calculated using the following formula:

$$SGMRS=\underset{i=1}{\sum }\left({\beta_{i}*\mathrm{Exp}}_{i}\right)$$

In this formula, *β* represented the coefficient of each critical SGMG when fitted by the concluding multivariate Cox regression model. *Exp* standed for the expression level of each essential SGMG. Furthermore, the optimal SGMRS cut-off value was settled by the function ‘surv_cutpoint’ of the R package ‘survminer’ with each group proportion ≥0.3. According to the optimal cut-off value, all patients were allocated into SGMRS low-risk or high-risk group. Eventually, to validate the efficacy of prognostic prediction, we illustrated the receiver operating characteristic (ROC) curves in validation sets of 1-, 2-, and 3-year survival rates and used the R package ‘time ROC’ to calculate the area under the ROC curve (AUC).

### Assessments of gene alterations and copy number variation

We obtained the data of gene alterations and copy number variation (CNVs) from the cBioPortal database (https://www.cbioportal.org/) for the TCGA cohort to assess the gene alterations and CNVs between different clusters and SGMRS risk groups. Subsequently, the R package ‘maftools’ was used to depict the different patterns of gene alterations and tumor mutation burdens (TMBs). Moreover, the CNV levels were represented as the Genomic Identification of Significant Targets in Cancer (GISTIC) score.

### Gene set enrichment analyses

In the section of gene set enrichment analyses, we used the R package ‘clusterProfiler’ to conduct the over-representation and gene set enrichment analysis (GSEA) to assess the differentially expressed genes (DEGs). Besides, we used the R package ‘limma’ to determine the DEGs between clusters and risk groups. DEGs were defined as those genes with |log2FC| > 0.5 and adjusted *p*-value < 0.05. In the GSEA, the DEGs were arranged according to their log2FC values and a Running Enrichment Score for each gene set was computed by adding 1/(number of DEGs) when a DEG was found in the gene set and subtracting 1/(number of DEGs) if not. Moreover, we converted the logFPKM matrix of genes to the pathway expression matrix using the R package ‘GSVA’. The differentially expressed pathways between clusters and risk groups were identified with the ‘limma’ package.

### Comprehensive characterization of tumor immune microenvironment based on serine and glycine metabolism

To explore the impact of serine and glycine metabolism on the tumor immune cells and immune microenvironment, we conducted multiple analyses to characterize the differences in the tumor immune microenvironment between different clusters and risk groups. Firstly, we applied the website of CIBERSORTx (https://cibersortx.stanford.edu/). Subsequently, we utilized the Estimation of Stromal and Immune Cells in Malignant Tumor issues using Expression data (ESTIMATE) to calculate the stromal, immune, and ESTIMATE scores in glioma, contributing to evaluating the infiltration of stromal and immune cells in the tumor microenvironment (Yoshihara et al., 2013). In this algorithm, the non-hematopoiesis-related genes that were differentially expressed between tumor cells and matched stromal cells separated by laser capture microdissection in multiple cancers were screened. The stromal related genes were selected from these genes. Besides, we also integrated the tumor purity data based on the ESTIMATE score and consensus purity estimation (CPE) previously published by D.Aran et al. (Aran et al., 2015). To identify the tumor immunological phenotype (TIP), we applied another previously published algorithm (Wang et al., 2021) to compute the TIP gene signature. According to the TIP gene signature, we could identify the immunological phenotype of tumor as either relatively ‘cold’ or ‘hot’ tumors. Additionally, the Tumor Immune Dysfunction and Exclusion (TIDE) suite (http://tide.dfci.harvard.edu/) was applied to predict potential response to therapy with ICIs.

### Nomogram construction based on SGMRS and other prognostic factors

To construct a nomogram that could effectively predict glioma patients' prognosis, we initially identified independent prognostic factors using univariate and multivariate Cox regression analyses. Firstly, the SGMRS, together with other potential prognostic factors, including age, gender, tumor grade, radiotherapy, chemotherapy, Karnofsky Performance Scale (KPS), isocitrate dehydrogenase (IDH) mutation, and 1p/19q codeletion, were enrolled into univariate Cox regression analysis. Then those prognostic factors with *p*-value < 0.05 in univariate Cox regression analysis entered the following multivariate analysis. Eventually, those prognostic factors with *p*-value < 0.05 in multivariate Cox regression analysis were determined as independent prognostic factors.

The nomograms were constructed based on these independent prognostic factors using the R package ‘rms’. To assess the efficacy of nomograms in the prediction of prognosis, we computed calibration curves for each nomogram.

### Statistical analyses

The R software (version 4.2.1) was used to perform the above bioinformatic analyses unless otherwise specified. For continuous variables, the Wilcoxon rank sum test was used to evaluate the differences between different clusters and risk groups. For categorical variables, the chi-square test was used to evaluate the differences. All the survival analyses were performed using the R package ‘survminer’. The differences between Kaplan-Meier (K-M) curves were tested by log-rank test. Univariate and multivariate Cox regression analyses were conducted using the ‘coxph’ function of the R package ‘survival’. The LASSO Cox regression analysis was performed using the R package ‘glmnet’. In linear regression analysis, the T Iterative Grubbs test was utilized to exclude the outliers.

### Ethic approval and data availability

The collection processes of clinical data and tumor samples were approved by the institutional review board of West China Hospital (No. 2018.569) following the 1964 Helsinki declaration and its later amendments. Besides, every patient signed written consent for collecting and using tumor tissue and clinical information. All the tumor tissue sequencing data from West China Hospital were available at the Genome Sequence Archive for Humans with accession code: HRA002839 (https://ngdc.cncb.ac.cn/gsa-human/s/JQssVoV1).

## Results

### Unsupervised consensus clustering analyses based on serine and glycine metabolism-related genes

**FIGURE 1:**
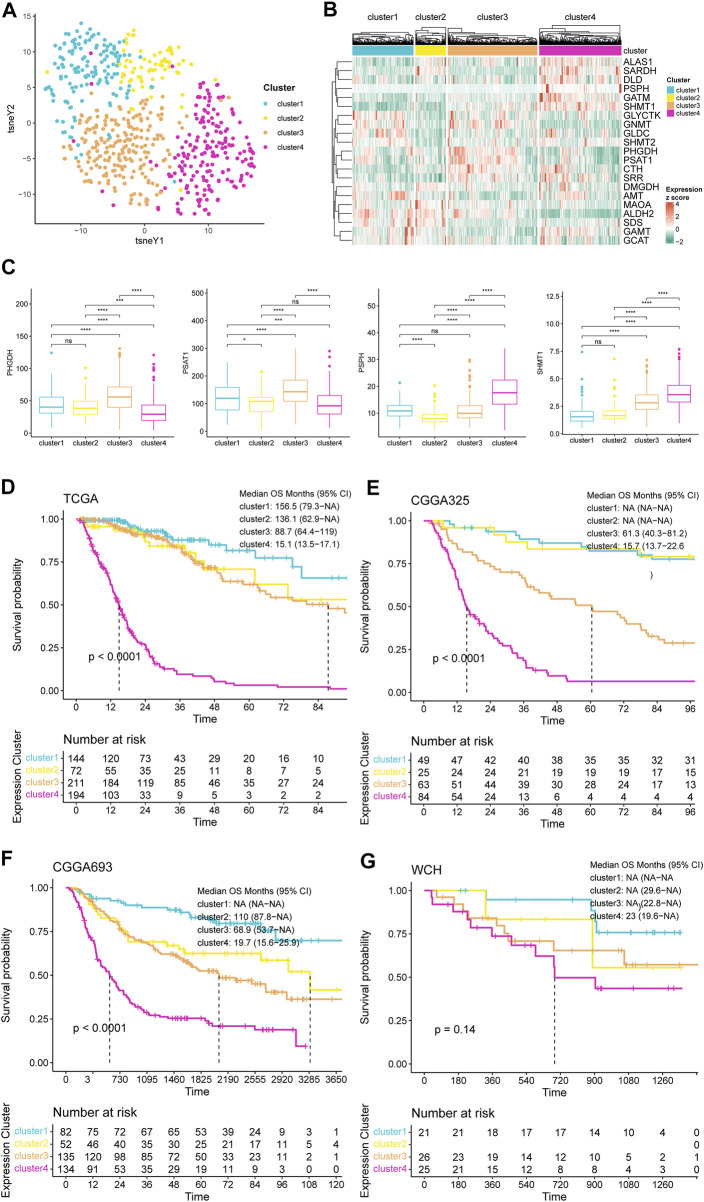
Clustering of gliomas based on expression pattern of SGMGs. **(A)** tSNE map for SGMGs expression patterns of four consensus clusters. **(B)** Heatmap for expression of 21 SGMGs based on four clusters. **(C)** The expression levels of PHGDH, PSAT1, PSPH, and SHMT1 among four clusters. **(D)** K-M curves based on four consensus clusters in **(D)** TCGA, **(E)** CGGA325, **(F)** CGGA693, and **(G)** WCH cohorts.

Based on the expression patterns of 21 serine and glycine metabolism-related genes (SGMGs), we performed an unsupervised consensus clustering analysis in patients of TCGA cohort. According to the clustering algorithm explained in Material and Method section, the delta area of CDF dropped significantly when k increased from 3 to 4, which suggest convergence of within-cluster similarity over between-cluster similarity with increased k over 4. Therefore, 4 was chosen to be the optimal number of clusters, and patients of TCGA cohort were classified into four consensus clusters (Supplementary Figure S1). The different expression patterns of SGMGs among these four clusters were illustrated using tSNE analysis (Figure 1A). Besides, the expression levels of four important genes involved in serine and glycine synthesis were illustrated (Figure 1B). Notably, cluster 3 significantly highly expressed PHGDH and PSAT1, and cluster 4 significantly highly expressed PSPH and SHMT1 (Figure 1C). The expression levels of all SGMGs in different clusters are illustrated in Supplementary Figure S1.

The survival analysis demonstrated that the prognosis of cluster 4 was overwhelmingly worse than the other three clusters in TCGA cohort (Figure 1D). Based on the naïve Bayes clustering classifier trained by the TCGA cohort, patients of the other three cohorts were also classified into four clusters. In survival analyses, the other three cohorts also exhibited the same trend (Figures 1E, F), indicating that the expression pattern of SGMGs was related to the prognosis of glioma patients even in independent glioma cohorts.

**FIGURE 2:**
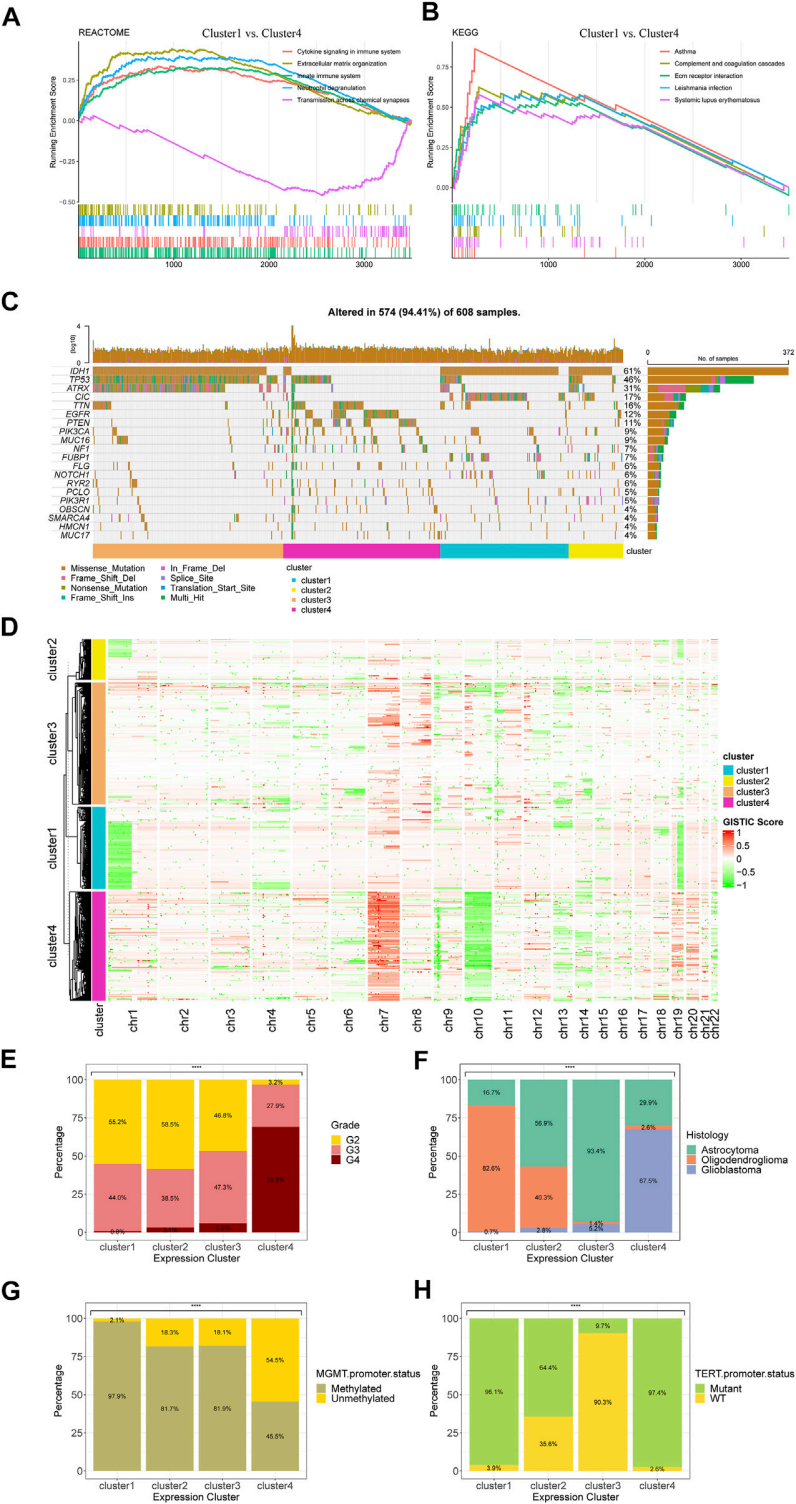
Functional enrichment and clinicopathological characteristics of the four consensus clusters in TCGA cohort. **(A)** Top five pathways with the highest NES in the REACTOME gene set between cluster 1 and cluster 4. **(B)** Top five pathways with the highest NES in the KEGG gene set between cluster 1 and cluster 4. **(C)** Top 20 mutated genes of the four consensus clusters. **(D)** Heatmap for copy number variations of the four clusters. **(E)** The differences in **(E)** tumor grade, **(F)** histological diagnosis, **(G)** MGMT promoter status, and **(H)** TERT promoter status among four clusters. *p < 0.05; **p < 0.01; ***p < 0.001; ****p < 0.0001.

To investigate the distinctive patterns of pathway alterations related to serine and glycine metabolism, we conducted functional enrichment analyses between cluster 1 and cluster 4, which showed the most differential SGMGs expression profiles and prognosis. In GSEA, the cytokine signaling in immune system pathway (NES = 2.739, adjusted *p*-value < 0.001) and the extracellular matrix organization pathway (NES = 3.165, adjusted *p*-value < 0.001) ranked among the top five REACTOME gene sets in the differentially expressed genes (DEGs) between cluster 1 and 4 (Figure 2A), suggesting the potential impact of serine and glycine metabolism on the tumor microenvironment and immunity. Besides, the extracellular matrix receptor interaction pathway (NES = 2.869, adjusted *p*-value <0.001) and the asthma pathway (NES = 2.983, adjusted *p*-value < 0.001) were also among the top 5 most significantly enriched Kyoto Encyclopedia of Genes and Genomes (KEGG) gene sets in the cluster 1/4 DEGs (Figure 2B), indicating the potential effect on inflammation and neurogenesis in glioma.

The results of gene mutation analysis revealed different gene mutation models of each cluster (Figure 2C). IDH1 mutation, a critical marker for diagnosis and prognosis of gliomas, was frequently observed in cluster 1, 2, and 3 but hardly occurred in cluster 4. Moreover, the mutation rates of TP53 and ATRX in cluster 3 were remarkably higher than the other 3 clusters. Moreover, most CIC mutations occurred in cluster 1. The analysis of CNVs also suggested distinctive characteristics among the four clusters. The gain of chromosome 7 and loss of chromosome 10 (+7/-10), which was recognized as a diagnostic marker for glioblastoma, predominantly occurred in cluster 4.1p/19q codeletion, which was indispensable for diagnosis of oligodendroglioma, mainly occurred in cluster 1, in line with the best prognosis of cluster 1. In clinicopathological features, the proportion of WHO grade 4 tumors grew from cluster 1 to cluster 4, which were characterized by glioblastomas and gliomas with unmethylated MGMT promoter, while TERT promoter wild-type tumors occupied the majority of cluster 3 gliomas, suggesting a potential connection between these tumors and alternative telomere lengthening (Figures 2E–H). Additionally, the differences in other clinicopathological features among these four clusters were also illustrated in Supplementary Figure S2.

### Analyses of immune cells infiltration and tumor microenvironment based on consensus clusters

**FIGURE 3:**
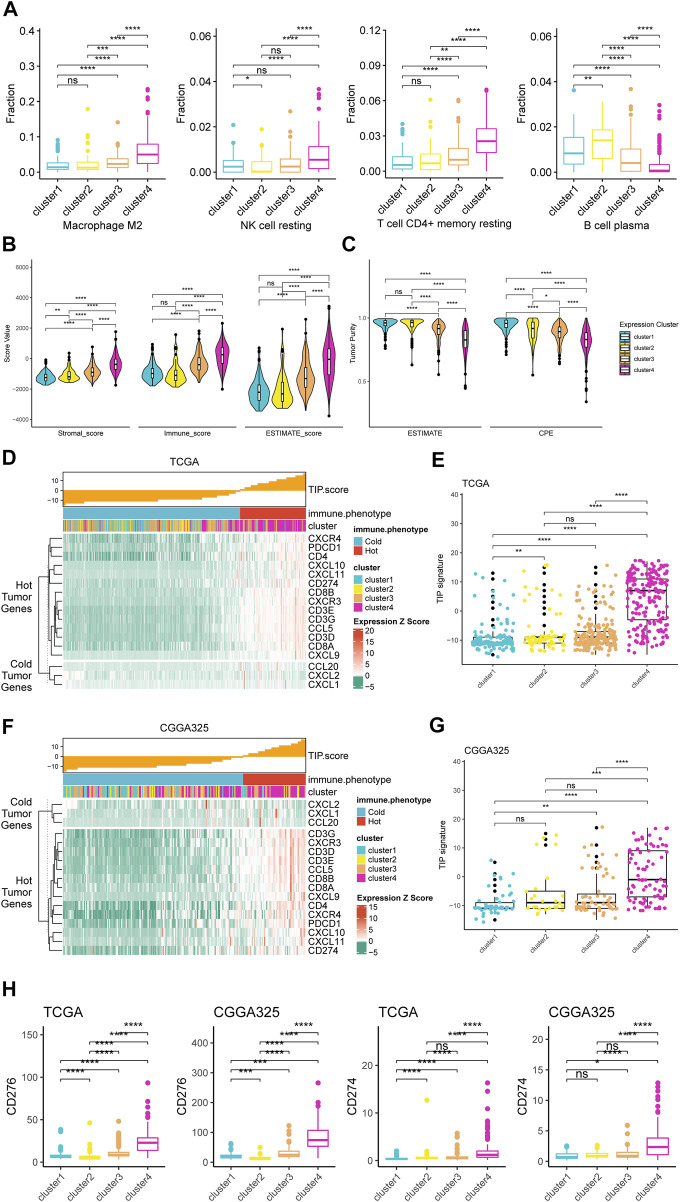
Different immunological landscapes of tumor microenvironment among four clusters. **(A)** Boxplots for infiltration fraction of four types of immune cells based on CIBERSORTx in TCGA cohort. **(B)** Differences in stromal, immune, and ESTIMATE scores among four clusters in TCGA cohort. **(C)** Difference in tumor purity among four clusters in TCGA cohort. **(D)** TIP score and related gene expression heatmap among four clusters in TCGA cohort. **(E)** Difference in TIP score among four clusters in TCGA cohort. **(F)** TIP score and related gene expression heatmap among four clusters in CGGA325 cohort. **(G)** Difference in TIP score among four clusters in CGGA325 cohort. **(H)** Differences in expression levels of CD274 and CD276 among four clusters in TCGA and CGGA 325 cohorts. *p < 0.05; **p < 0.01; ***p < 0.001; ****p < 0.0001.

Based on the consensus clusters, we performed comprehensive analyses to explore the impact of serine and glycine metabolism on the immune cells and immune microenvironment in glioma. The analyses of immune cell infiltration in the tumor microenvironment revealed that there were more abundant M2 macrophages, resting NK cells, and resting memory CD4^+^ T cells but fewer plasma B cells in the tumor microenvironment of cluster 4 (Figure 3A). Besides, the calculation of stromal, immune, and ESTIMATE scores based on the ESTIMATE algorithm demonstrated that cluster 4 had remarkably higher scores than the other three clusters. Higher stromal, immune, and ESTIMATE scores represented for more stromal cells and more immune infiltration in the tumor microenvironment. In comparison, cluster 3 gliomas also had significantly higher scores than cluster 1 and 2, suggesting that these tumor microenvironment-related scores were related to serine and glycine metabolism and prognosis in glioma (Figure 3B). Furthermore, for the analysis of tumor purity, cluster 4 was manifested with significantly lower tumor purity than other clusters, indicating a more complex tumor microenvironment of cluster 4 (Figure 3C). Moreover, based on the computation of the TIP score, cluster 4 was demonstrated with higher expression of genes related to the ‘hot’ immunological phenotype of tumor than other clusters in TCGA cohort (Figure 3D). The resulting TIP score of cluster 4 was significantly higher than other clusters, suggesting cluster 4 could be a relatively ‘hotter’ tumor compared to those in other clusters (Figure 3E). These findings were also validated in CGGA325 cohort (Figure 3F, G), suggesting a robust association between the expression of serine/glycine metabolism-related genes and the immune landscape of gliomas. Additionally, analyses of markers related to immunotherapy revealed that expression levels of CD274 (PD-L1) and CD276 (B7-H3), which were essential targets for immunotherapy, expressed at remarkably higher levels in cluster 4 compared to other clusters in TCGA and CGGA325 cohorts (Figure 3H). Combined results of TIP score and the expression levels of immunotherapy-related targets demonstrated that cluster 4, which exhibited ‘hotter’ immunological phenotype and expressed more immunotherapy-related targets, might be more likely to response to immunotherapy than patients of other clusters.

### Construction and validation of serine and glycine metabolism-related genes risk signature

**FIGURE 4:**
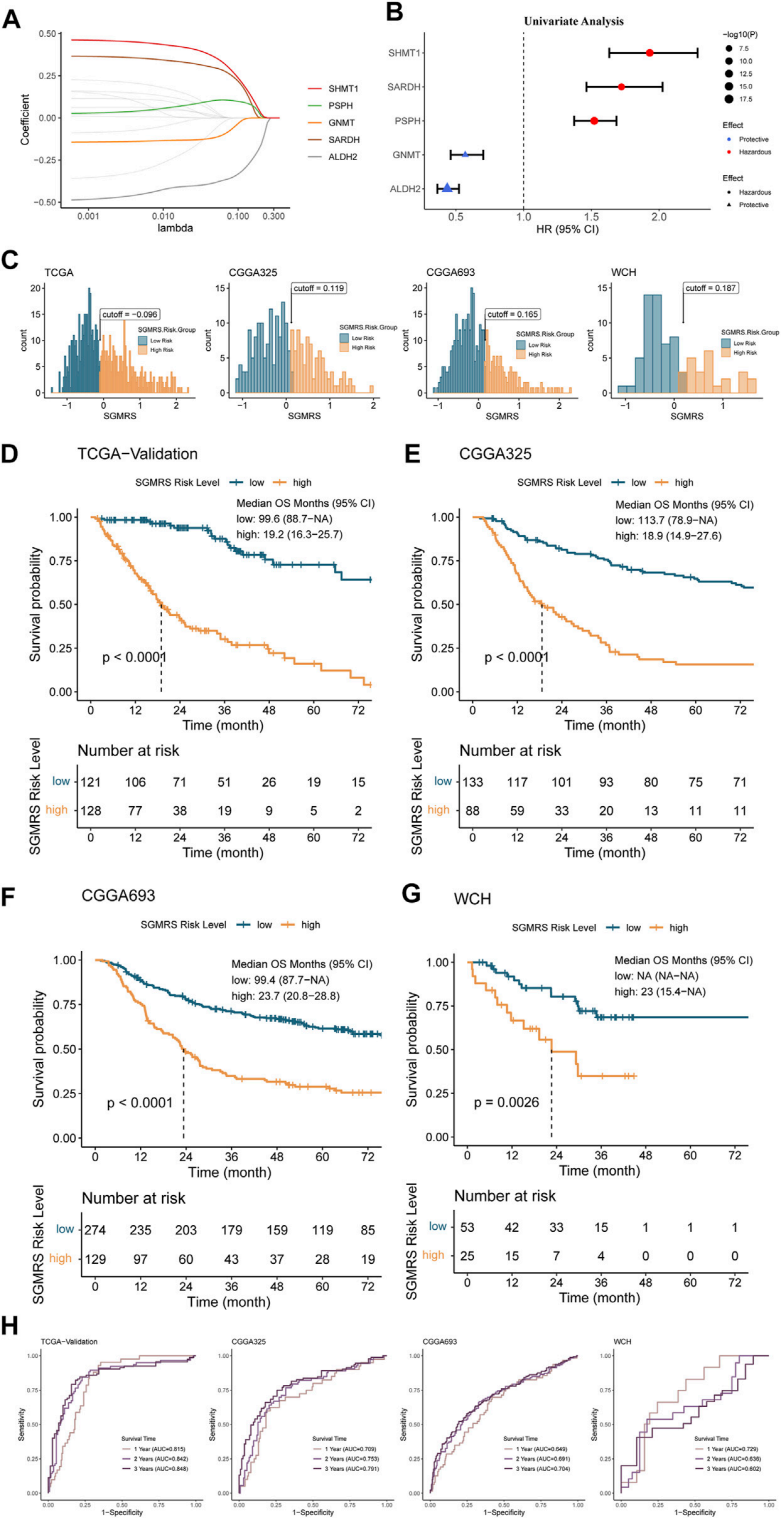
Construction of SGMRS and its relationship with prognosis. **(A)** Average of coefficients of five critical SGMGs in the LASSO Cox regression at each lambda value. **(B)** The prognostic effect of each critical SGMG in glioma. **(C)** Optima cutoff value of SGMRS in all four cohorts. **(D)** K-M curves of the **(D)** TCGA, **(E)** CGGA325, **(F)** CGGA693, and **(G)** WCH cohorts based on SGMRS high- and low-risk groups. **(H)** ROC curves and matched AUC of 1-, 2-, 3-year survival rate in all four cohorts.

In this section, we filtered the 21 SGMGs with the LASSO Cox regression in training set to identify critical genes for the construction of serine and glycine metabolism-related genes risk signature (SGMRS). Five SGMGs, including SHMT1, PSPH, GNMT, SARDH, and ALDH2, were identified as critical genes for the construction of SGMRS (Figure 4A), and the formula of the SGMRS was derived by fitting a final multivariate Cox regression model to the expression of the 5 critical SGMGs in the training dataset. The SGMRS was calculated using the following formula:

#### 0.505*SARDH+0.243*SHMT1-1.77e-4*PSPH-0.050*ALDH2-0.209*GNMT

Further univariate analysis demonstrated that SHMT1, SARDH, and PSPH were hazardous prognostic factors for glioma (Figure 4B). GNMT and ALDH2 were protective factors for glioma (Figure 4B). Moreover, to validate these results, we obtained representative immunohistochemical staining for SARDH and PSPH from the Human Protein Atlas (https://www.proteinatlas.org/) (Pontén et al., 2008). The staining figures revealed that the protein levels of SARDH and PSPH were higher in high-grade gliomas compared to low-grade gliomas (Supplementary Figure 3A-B), which was consistent with the results of the univariate analysis. Subsequently, the ‘surv_cutpoint’ algorithm was used to identify the optimal SGMRS cut-off for all these four cohorts, and the patients were classified into SGMRS high- and low-risk groups based on this cut-off (Figure 4C). Further survival analyses revealed that the patients in SGMRS high-risk group had an enormously poorer prognosis than low-risk group in the TCGA validation cohort (Figure 4D), which was also confirmed by the other three cohorts (Figures 4E,F). We also conducted ROC analyses to examine the efficacy of SGMRS to predict survival rates at 1, 2, and 3 years. AUCs of the ROC curves of SGMRS in TCGA validation cohort at 1, 2, and 3 years was 0.815, 0.842, and 0.848, endorsing the effectiveness of SGMRS on prognosis prediction (Figure 4H). In the other three cohorts, the performances of SGMRS on prognosis prediction were similar (Figure 4H).

**FIGURE 5:**
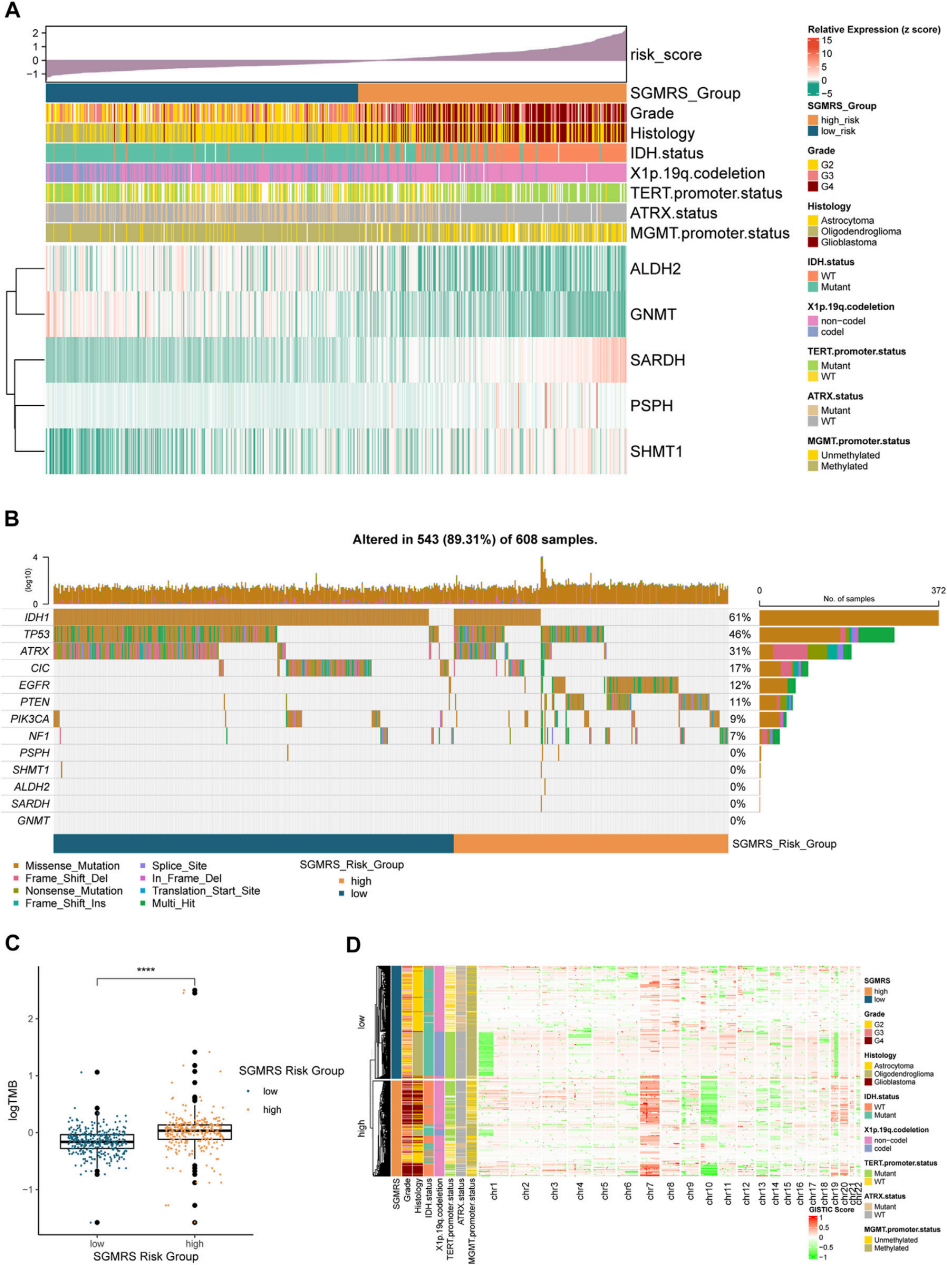
Clinicopathological features of SGMRS risk groups. **(A)** Expression level of five critical SGMGs and its relationship with clinicopathological features. **(B)** Gene mutations of five critical SGMGs and top eight frequently mutated genes in gliomas ordered by SGMRS risk groups. **(C)** Difference in tumor mutation burden between SGMRS high- and low-risk groups. **(D)** Copy number variation and its relationship with clinicopathological features ordered by SGMRS risk groups. *p < 0.05; **p < 0.01; ***p < 0.001; ****p < 0.0001.

To illustrate the expression pattern of these five critical SGMGs, we aligned a heatmap of the expression level of each patient in order of SGMRS. Besides, the clinicopathological features, including tumor grade, histology diagnosis, IDH status, 1p/19q codeletion, TERT promoter status, ATRX status, and MGMT promoter status, were also integrated (Figure 5A). As for the analysis of gene mutations, the SGMRS high-risk group manifested with a lower incidence of IDH1 and TP53 mutation (Figure 5B) and a higher incidence of EGFR and PTEN mutation. Furthermore, the tumor mutation burden (TMB) analysis between SGMRS high- and low-risk groups revealed a significantly higher TMB in high-risk groups (Figure 5C). Additionally, the analysis of CNVs demonstrated that most of chromosome +7/-10 occurred in SGMRS high-risk group (Figure 5D), and most of the 1p/19q codeletion occurred in the low-risk group.

### Functional enrichment analyses based on SGMRS risk groups

**FIGURE 6:**
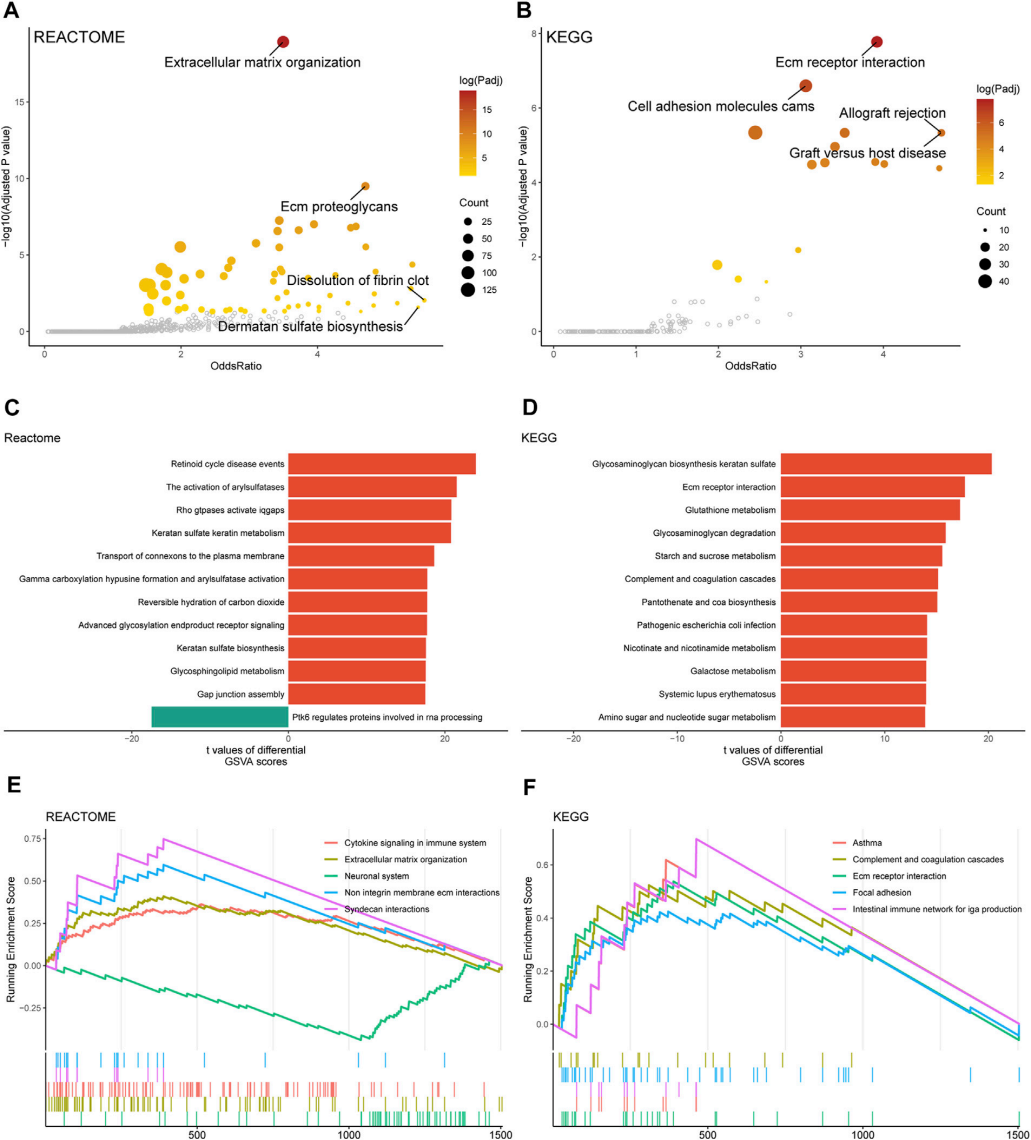
Functional enrichment analyses between two SGMRS risk groups. **(A)** Pathways with high confidence and odds ratio in REACTOME gene sets. **(B)** Pathways with high confidence and odds ratio in KEGG gene sets. **(C)** Top 12 pathways in REACTOME gene set with the highest GSVA scores. **(D)** Top 12 pathways in KEGG gene set with the highest GSVA scores. **(E)** Top five pathways in REACTOME gene set with the highest normalized enrichment scores. **(F)** Top five pathways in KEGG gene set with the highest normalized enrichment scores.

In this section, we performed multiple functional enrichment analyses to investigate the pathway alterations in different SGMRS risk groups. The extracellular matrix organization of REACTOME gene sets and the extracellular matrix receptor interaction of KEGG gene sets were identified with high odds ratio and *p*-value between high- and low-risk groups (Figures 6A,B). The retinoid cycle disease events pathway was listed in the top 12 dysregulated pathways in the REACTOME gene set (Figure 6C). The glutathione metabolism pathway, which was regulated by serine and glycine synthesis (Geeraerts et al., 2021), was listed in the top 12 pathways with highest GSVA scores in the KEGG gene set (Figure 6D). Besides, the cytokine signaling in immune system pathway (NES = 2.568, adjusted *p*-value <0.001) and extracellular matrix organization pathway (NES = 2.718, adjusted *p*-value <0.001) of REACTOME gene sets were ranked in the top five pathways in comparison between two SGMRS risk groups using GSEA (Figure 6E). The complement and coagulation cascades pathway (NES = 2.223, adjusted *p*-value <0.001) and the focal adhesion pathway (NES = 2.354, adjusted *p*-value <0.001) of KEGG gene sets were ranked in the top five (Figure 6F).

### Construction of nomograms based on SGMRS to predict prognosis in glioma patients

**FIGURE 7:**
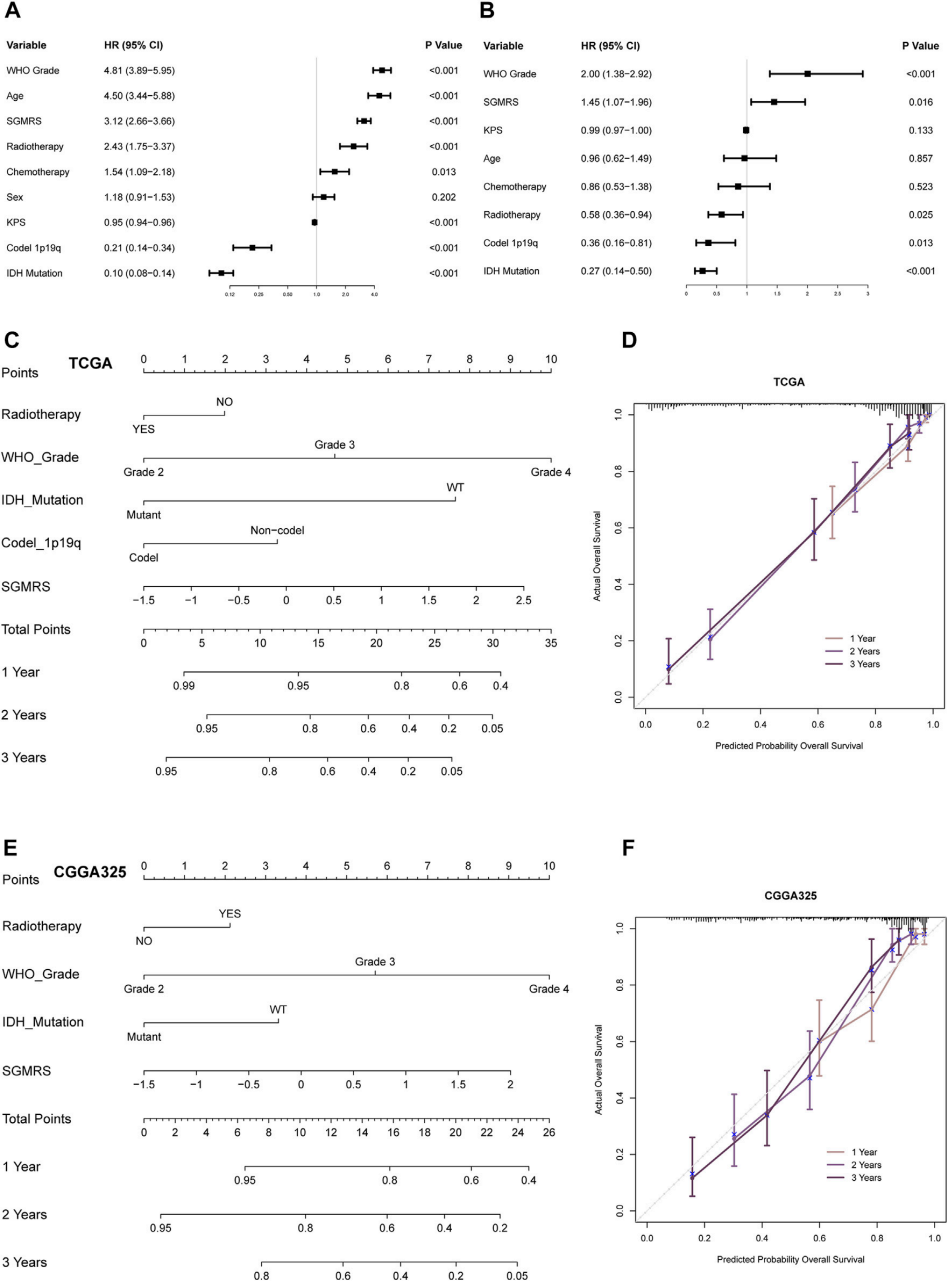
Prognostic value of SGMRS and construction of SGMRS-based nomograms. **(A)** Univariate analysis of potential prognostic factors in glioma. **(B)** Multivariate analysis to identify independent prognostic factors in glioma. **(C)** Nomogram of 1-, 2-, and 3-year survival rate of glioma patients in TCGA cohort. **(D)** Calibration plots for the nomogram of TCGA cohort. **(E)** Nomogram of 1-, 2-, and 3-year survival rate of glioma patients in CGGA325 cohort. **(F)** Calibration plots for the nomogram of CGGA325 cohort.

We firstly conducted univariate and multivariate Cox regression analyses to identify independent prognostic factors for the subsequent construction of nomograms. The SGMRS, together with other potential prognostic factors, including tumor grade, patient age, radiotherapy, chemotherapy, sex, KPS, 1p/19q codeletion, and IDH mutation, were enrolled into univariate Cox regression analysis in TCGA cohort (Figure 7A). Subsequently, those prognostic factors (*p*-value < 0.05 in univariate analysis) were enrolled into multivariate Cox regression analysis. Eventually, the SGMRS, together with tumor grade, radiotherapy, 1p/19q codeletion, and IDH mutation, were identified as independent prognostic factors in glioma (*p*-value < 0.05, Figure 7B). These factors were utilized to construct a nomogram to achieve individualized survival rate prediction (Figure 7C). The corrected C-index of this nomogram based on TCGA cohort was 0.848. This nomogram’s efficacy in predicting the prognosis of glioma patients was validated by the 1-, 2-, and 3-year calibration curves (Figure 7D). For the CGGA325 and cohort, the corrected C-index of the nomogram was 0.765 (Figure 7E). For the CGGA693 cohort and WCH cohort, it is 0.772 and 0.696 respectively. The 1-, 2-, and 3-year calibration curves derived from CGGA325 dataset also endorsed performance of the nomogram (Figure 7F).

### Correlation of SGMRS with immune cells and immune microenvironment

**FIGURE 8:**
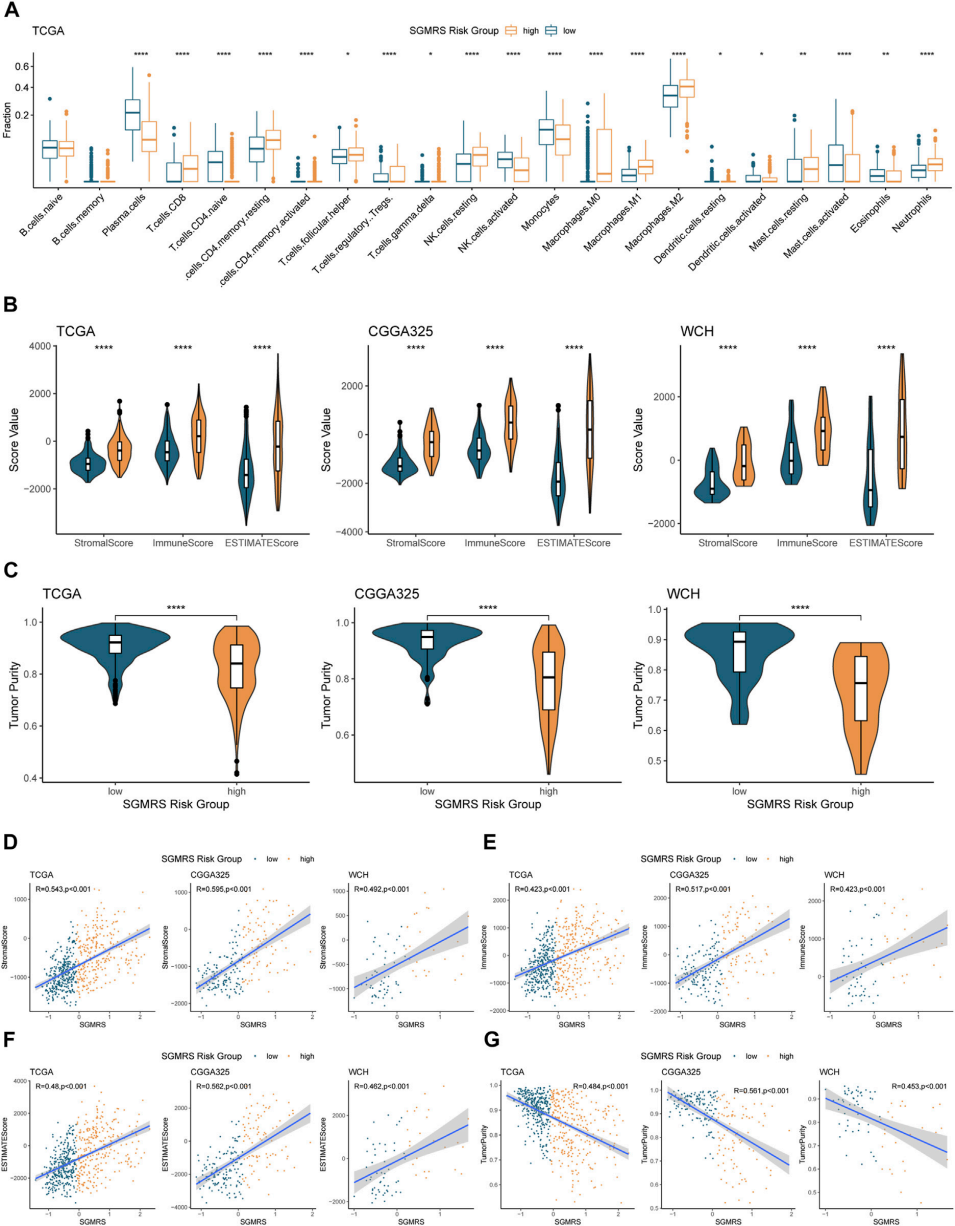
Analyses on immune landscapes of tumor microenvironment between SGMRS high- and low-risk groups. **(A)** Boxplot for the estimated infiltration fraction of 22 types of immune cells in tumors. **(B)** Differences in the stromal, immune, and ESTIMATE scores between two risk groups in TCGA, CGGA325, and WCH cohorts. **(C)** Differences in tumor purity between two risk groups in TCGA, CGGA325, and WCH cohorts. **(D)** Analyses of correlations of SGMRS with the **(D)** stromal score, **(E)** immune score, **(F)** ESTIMATE score, and **(G)** tumor purity in TCGA, CGGA325, and WCH cohorts. *p < 0.05; **p < 0.01; ***p < 0.001; ****p < 0.0001.

To investigate the connection between SGMRS and the immune landscape of gliomas, we performed comprehensive analyses to elucidate the correlation between SGMRS and multiple immunity-related indexes. Firstly, we computed the infiltration fraction of 22 types of immune cells in the tumor microenvironment using the CIBERSORTx algorithm. The results revealed that gliomas of SGMRS high-risk group harbored more macrophages (including M0, M1, and M2), resting NK cells, and resting memory CD4^+^ T cells infiltrated into the tumor microenvironment, and fewer plasma cells and activated NK cells (Figure 8A), depicting distinctive immune cell infiltration models between SGMRS high- and low-risk groups. Subsequently, we utilized the ESTIMATE algorithm to analyze immune-related scores and tumor purity. The SGMRS high-risk group manifested with higher stromal, immune, and ESTIMATE scores compared to the low-risk group in TCGA, CGGA325, and WCH cohorts (Figure 8B), indicating a significantly more complex tumor microenvironment in gliomas with higher SGMRS. The analysis of tumor purity also confirmed that gliomas of the high-risk group had remarkably lower tumor purity than those of the low-risk group, which was in accordance with the results of immune-related scores (Figure 8C). Further correlation analysis confirmed that the stromal score, immune score, and ESTIMATE score were strongly positively correlated with the value of SGMRS in these three cohorts (Figures 8D–F). The tumor purity was negatively correlated with the value of SGMRS in these three cohorts (Figure 8G).

**FIGURE 9:**
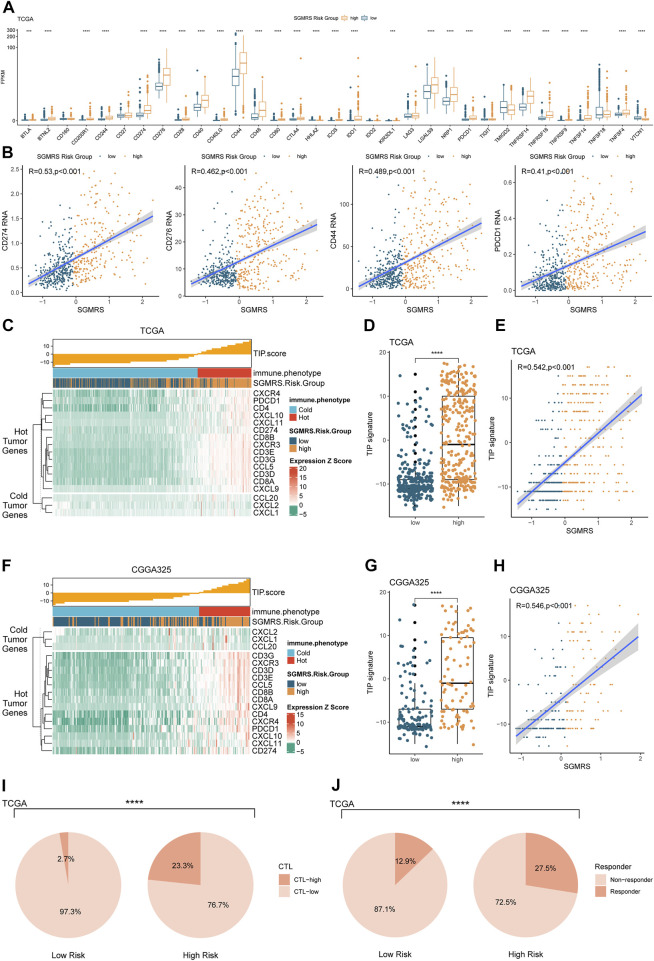
Differences in expression of immunotherapy-related genes, immunological phenotype, and response to ICIs between two SGMRS risk groups. **(A)** Boxplot for the expression level of 33 immunotherapy-related genes in two risk groups in TCGA cohort. **(B)** Analyses of correlations between SGMRS and the expression levels of CD274, CD276, CD44, and PD-1 in TCGA cohort. **(C)** Analysis of TIP score and related gene expression levels ordered by SGMRS in TCGA cohort. **(D)** Difference in TIP score between two risk groups in TCGA cohort. **(E)** Analysis of correlation between SGMRS and TIP score in TCGA cohort. **(F)** Analysis of TIP score and related gene expression levels ordered by SGMRS in CGGA325 cohort. **(G)** Difference in TIP score between two risk groups in CGGA325 cohort. **(H)** Analysis of correlation between SGMRS and TIP score in CGGA325 cohort. **(I)** Difference in proportion of patients with high cyto-toxic T lymphocytes infiltration between two risk groups in TCGA cohort. **(J)** Difference in proportion of patients with predictive response to immune checkpoint inhibitors between two risk groups in TCGA cohort.

To explore potential applications of SGMRS in the guidance of immunotherapy, we analyzed the relationship between multiple immunotherapy-related markers and SGMRS. In gliomas of SGMRS high-risk group, the expression levels of CD274 (PD-L1), CD276 (B7-H3), and CD279 (PD-1) were remarkably higher compared to the low-risk group in TCGA cohort (Figure 9A). In the CGGA325 cohort, this result was also confirmed (Figure 9B), indicating that gliomas with high SGMRS would overexpress multiple targets for immunotherapy. Furthermore, to validate the potential ability of SGMRS to direct the use of immunotherapy, we calculated the TIP score to identify the relationship between the immunological phenotype and SGMRS in glioma. The result demonstrated that gliomas of SGMRS high-risk group would highly express immunological ‘hot’ tumor genes (Figure 9C) in TCGA cohort. And the TIP scores of gliomas in the high-risk group were enormously higher than low-risk group (Figure 9D). Correlation analysis confirmed the positive correlation between TIP score and SGMRS (Figure 9E). These findings were also validated in the CGGA325 cohort (Figures 9F–H). Additionally, the analysis of cytotoxic T cells (CTLs) revealed that the gliomas of SGMRS high-risk group harbored more CTLs infiltration compared to the low-risk group in TCGA and CGGA325 cohort (Figure 9I). It is also demonstrated that patients of the high-risk group would respond better to immune checkpoint inhibitors compared to low risk in TCGA cohort (Figure 9J). Most of these findings can be validated in other cohorts (Supplementary Figure S4).

## Discussion

As one of the most lethal cancer, glioma causes more than 200 thousand deaths worldwide every year (Sung et al., 2021). Due to its heavy burden and direct threats to human health, countless researchers devoted themselves to exploring novel therapies to improve the prognosis of glioma patients. However, there is hardly inspiring breakthrough in the field of glioma, especially for glioblastoma, which accounts for 50% of gliomas and presented with only a median overall survival of 22 months after the complete treatment process, including surgery resection, radiotherapy, chemotherapy, and even tumor treating field (Stupp et al., 2005; Stupp et al., 2017). Immunotherapy, emerging under the spotlight as a novel therapy for cancers, has been proven effective in multiple types of cancer (Eggermont et al., 2018; Gandhi et al., 2018; Choueiri et al., 2021b; Cortes et al., 2022). Hence, several studies have concentrated on the potential therapeutic effects of immunotherapy in glioma. However, almost all these attempts at the application of immunotherapy failed to improve the overall survival of glioma patients in phase 3 clinical trials (Weller et al., 2017; Wakabayashi et al., 2018; Reardon et al., 2020; Lim et al., 2022; Omuro et al., 2022). The blood-brain barrier (BBB), which functions to block most peripheral immune cells out of the central nervous system (CNS), was recognized as an important reason for these failures. However, inspiringly, a novel lymphatic pathway permitting antigen-presenting cells to escape from CNS was introduced in recent years (Louveau et al., 2015). Further research proved that lymphocytes outside CNS could be primed by these antigen-presenting cells and then infiltrate into the brain and execute immune responses (Lim et al., 2018). These studies suggest that brain is not a closed area for applications of immunotherapy. If we can further investigate and understand the mechanisms of immune cell infiltrations and reshaped immune landscapes of the tumor microenvironment, immunotherapy might become another robust weapon for us to fight against glioma. Therefore, our present study devoted to investigating the underlying mechanisms of the unique immune landscape of glioma, aiming to provide potential help to the application of immunotherapy.

In the field of tumor immunity, the relationship between unique metabolic patterns and immunological characteristics of tumors has become an attractive topic (Xia et al., 2021). Many studies have suggested that serine and glycine metabolism has critical effects on cancers (DeBerardinis, 2011; DeBerardinis and Chandel, 2016). As two non-essential amino acid, cells can gain serine and glycine through intracellular synthesis and uptake from the environment (de Koning et al., 2003; Sullivan and Vander Heiden, 2017). The upregulation of serine and glycine synthesis has been observed in many cancers (Kim et al., 2015; Liao et al., 2019b). As a side-branch of glycolysis, serine and glycine synthesis was tightly regulated by the activity of glycolysis. Due to the Warburg effect, cancer cells could fulfil the requirement of glycolytic intermediates in the synthesis of serine and glycine through activated aerobic glycolysis (DeBerardinis and Chandel, 2020). Upregulating the activity of M2 isoform of pyruvate kinase (PKM2), an enzyme functioned to catalyze conversion of phosphoenolpyruvate into pyruvate, can restrict 3-PG, the initial compound of serine and glycine synthesis, channeling into serine and glycine synthesis (Chaneton et al., 2012). In cancer cells, activation of PKM2 can reduce the synthesis of serine and glycine and render cancer cells dependent on uptake from environment (Kung et al., 2012). On the other way, restriction of dietary serine and glycine, which functioned to decrease serine and glycine uptake from environment, can reduce tumor growth (Maddocks et al., 2013; Gravel et al., 2014). But this effect was alleviated in those cancer models with upregulated serine and glycine synthesis, suggesting that the synthesis of serine and glycine can compensate the lack of uptake from environment (Maddocks et al., 2017). Therefore, the simultaneous application of inhibiting serine and glycine synthesis and uptake exhibited a promising effect and called for more studies.

Moreover, in glioma, the concentration of glycine was also proved with a positive correlation with aggressiveness (Tiwari et al., 2020). Furthermore, serine and glycine were manifested as immunosuppressive metabolites (He et al., 2019). Cancer cells can overproduce abundant serine and glycine, which delivers robust immunosuppressive effects and might contributes to the immune evasion of cancer cells (Hanahan and Weinberg, 2011). Extracellular serine can suppress the function of macrophages and neutrophils (He et al., 2019). High activity of PHGDH would promote macrophages to differentiate into M2-like (Wilson et al., 2020). Hence, investigating the relationship between serine and glycine metabolism and the immune landscape of glioma may contribute to the application of immunotherapy.

In the present study, to explore the relationship between SGMGs and clinicopathological features and the immune landscape of gliomas, we firstly classified all patients into four consensus clusters based on their distinctive expression patterns of SGMGs. Compared to the other clusters, gliomas in cluster 4 expresses significantly higher levels of PSPH and SHMT1 which were known culprits of aberrant serine and glycine production in malignant cancers (Geeraerts et al., 2021). Additionally, the strong immunohistochemistry signal of PSPH in high grade gliomas presented as an example that the dysregulated SMGMs could be used as pathological biomarkers to identify the most aggressive gliomas (Supplementary Figure S3). Among these four clusters, different clinicopathological features and prognosis patterns were depicted. Furthermore, the incidences of gene alterations also differed among these four clusters. For instance, IDH mutation, a critical diagnostic and prognostic marker for glioma would lead to abnormal tricarboxylic acid (TCA) cycle (Yan et al., 2009; Pirozzi and Yan, 2021). Besides, the serine and glycine synthesis pathway was reported to provide approximately 50% of the total anaplerotic flux of glutamine into the TCA cycle (Possemato et al., 2011), indicating potential interaction between serine and glycine metabolism and IDH mutation. Nevertheless, even with potential interaction with other prognostic factors, the SGMRS was still proved as an independent prognostic factor in multivariate analysis, which included SGMRS and other potential prognostic factors, indicating the satisfactory potential of SGMRS as a prognostic factor.

After filtering SGMGs, five SGMGs were identified as critical genes for the prognosis of glioma, suggesting the strong interaction between these five genes and glioma. For example, phosphoserine phosphatase (PSPH), an essential enzyme of serine and glycine metabolism, catalyzes the dephosphorylation of phosphoserine to serine. In multiple cancers, PSPH promotes tumor growth and metastasis (Liao et al., 2019a; Rawat et al., 2021). In our study, the hazardous effect of PSPH was illustrated. Serine hydroxymethyl transferase 1 (SHMT1) is a critical enzyme that converts serine to glycine (Hebbring et al., 2012). Upregulation of SHMT1 would increase the concentration of glycine. Several studies have found that SHMT1 can promote tumor growth and progression (Pandey et al., 2014; Gupta et al., 2017). The activity of SHMT1 was strongly negatively correlated with the overall survival in both clustering analysis and SGMRS analysis, which was accordance with previous study and endorsed the critical role of SHMT1 on the prognosis of glioma patients. Compared to other three essential enzymes of serine and glycine synthesis, SHMT1 showed significantly stronger correlation with prognosis both in consensus clustering analysis and in SGMRS analysis, suggesting that SHMT1 was the essential enzyme of serine and glycine synthesis to regulate the malignancy of glioma. Besides, glycine N-methyltransferase (GNMT) catalyzes the methylation of glycine to form sarcosine (Yeo and Wagner, 1994), which might decrease glycine concentration in the tumor. GNMT has been proven to have tumor suppression function in hepatocellular carcinoma (Chen et al., 1998). However, there is no study to elucidate the effects of GNMT in glioma. Our study suggested the protective effects of GNMT in glioma, inspiring further research on it.

Further analyses of immune cell infiltration and immune landscapes depicted the relationship between serine and glycine metabolism and the immune microenvironment of glioma. The CIBERSORTx analyses estimated the infiltration fraction of multiple types of immune cells. The results demonstrated that the infiltration of many immune cells was correlated with SGMRS. For example, the infiltration of M2 macrophages into the tumor microenvironment was strongly positively correlated with SGMRS. Circulating monocytes and neighboring macrophages can be recruited by tumor cells and then infiltrated into the tumor microenvironment. Subsequently, these macrophages were polarized from M1-like to M2-like, forming tumor-associated macrophages (TAMs) (Anderson et al., 2021). TAMs can synthesize cytokines to suppress the function of T lymphocytes and upregulated immunosuppressive surface proteins (Curiel et al., 2004; Colombo and Piconese, 2007; Yang and Zhang, 2017). These immunosuppressive functions of TAMs became an important reason for the immune evasion of tumors. The correlation between high SGMRS and high infiltration of TAMs suggests the role of serine and glycine metabolism in immune evasion, inspiring that serine and glycine metabolism could be another target to suppress immune evasion of glioma. The expression levels of multiple immunotherapy-related genes, including PD-1 and PD-L1, were also strongly positively correlated with SGMRS. The serine and glycine synthesis was also reported to induce macrophages to overexpress PD-L1 by promoting the release of IL-1β (Su et al., 2018; Rodriguez et al., 2019; Yu et al., 2019), according to our study. Additionally, higher SGMRS was correlated with immunological ‘hotter’ features and more potential responders to ICIs. These findings suggested the potential ability of SGMRS to predict the expression of targets for immunotherapy and the consequent ability to guide the selection and use of immunotherapy in glioma.

Although comprehensive analyses were conducted in our present study, there are still some limitations. First, protocols used for data preprocessing and sequencing were different among these four cohorts. Next, compared to metabolic and proteomic data, the abundance of public RNA-sequencing datasets allows more robust analysis and validation of the results in multiple independent cohorts. However, the results derived from transcriptome analysis as performed here would be still more impactful if validated in future experiments. Besides, all the analyses and related genes were about serine and glycine metabolism, in other word, in the scope of pharmacodynamics of serine and glycine. The disposition of serine and glycine in different organs or tissues might also influence their effects, which remains to be explored. In addition, due to lack of transcriptomic data from gliomas patients receiving immunotherapy, the implications of our findings are confined to estimated ICI responses rather than actual response. The application of the prediction results should be evaluated with a clinical study design. Finally, the underlying mechanism of how serine and glycine metabolism impacted immune cell infiltration and the immune landscape remains unclear and calls for further investigation.

## Conclusion

In conclusion, we demonstrated that expression patterns of SGMGs were closely related to clinicopathological features, immune cell infiltration, and the immune landscape of glioma. A novel serine and glycine metabolism assessment score system, SGMRS, exhibited with robust ability to predict the prognosis of glioma patients. Besides, higher SGMRS, standing for more glycine synthesis and less glycine catabolism, predicts more immune cells infiltration, a more complex tumor microenvironment, and more expression of targets for immunotherapy, endorsing the application of SGMRS to guide the choice and use of immunotherapy in glioma.

## Data Availability

The datasets presented in this study can be found in online repositories. The names of the repository/repositories and accession number(s) can be found in the article/Supplementary Material.
