# Supplementary material for: Serine and glycine metabolism-related gene expression signature stratifies immune profiles of brain gliomas, and predicts prognosis and responses to immunotherapy
Source: Front Pharmacol. 2022 Nov 17;13:1072253. doi: 10.3389/fphar.2022.1072253 (PMC9712738; doi:10.3389/fphar.2022.1072253)
Supplement: Supplementary file 1 [file Table1.DOCX]

| geneid | genesymbol | genename | name | Inclusion (Y:YES, N:NO) |
| --- | --- | --- | --- | --- |
| 189 | AGXT | alanine--glyoxylate aminotransferase | Serine--pyruvate aminotransferase | N |
| 211 | ALAS1 | 5'-aminolevulinate synthase 1 | 5-aminolevulinate synthase, nonspecific, mitochondrial | Y |
| 217 | ALDH2 | aldehyde dehydrogenase 2 family member | Aldehyde dehydrogenase, mitochondrial | Y |
| 275 | AMT | aminomethyltransferase | Aminomethyltransferase, mitochondrial | Y |
| 1491 | CTH | cystathionine gamma-lyase | Cystathionine gamma-lyase | Y |
| 1738 | DLD | dihydrolipoamide dehydrogenase | Dihydrolipoyl dehydrogenase, mitochondrial | Y |
| 1757 | SARDH | sarcosine dehydrogenase | Sarcosine dehydrogenase, mitochondrial | Y |
| 2593 | GAMT | guanidinoacetate N-methyltransferase | Guanidinoacetate N-methyltransferase | Y |
| 2617 | GARS1 | glycyl-tRNA synthetase 1 | Glycine--tRNA ligase | N |
| 2628 | GATM | glycine amidinotransferase | Glycine amidinotransferase, mitochondrial | Y |
| 2731 | GLDC | glycine decarboxylase | Glycine dehydrogenase [decarboxylating], mitochondrial | Y |
| 4128 | MAOA | monoamine oxidase A | Amine oxidase [flavin-containing] A | Y |
| 5723 | PSPH | phosphoserine phosphatase | Phosphoserine phosphatase | Y |
| 6301 | SARS1 | seryl-tRNA synthetase 1 | Serine--tRNA ligase, cytoplasmic | N |
| 6470 | SHMT1 | serine hydroxymethyltransferase 1 | Serine hydroxymethyltransferase, cytosolic | Y |
| 6472 | SHMT2 | serine hydroxymethyltransferase 2 | Serine hydroxymethyltransferase, mitochondrial | Y |
| 10993 | SDS | serine dehydratase | L-serine dehydratase/L-threonine deaminase | Y |
| 23464 | GCAT | glycine C-acetyltransferase | 2-amino-3-ketobutyrate coenzyme A ligase, mitochondrial | Y |
| 26227 | PHGDH | phosphoglycerate dehydrogenase | D-3-phosphoglycerate dehydrogenase | Y |
| 27232 | GNMT | glycine N-methyltransferase | Glycine N-methyltransferase | Y |
| 29958 | DMGDH | dimethylglycine dehydrogenase | Dimethylglycine dehydrogenase, mitochondrial | Y |
| 29968 | PSAT1 | phosphoserine aminotransferase 1 | Phosphoserine aminotransferase | Y |
| 63826 | SRR | serine racemase | Serine racemase | Y |
| 132158 | GLYCTK | glycerate kinase | Glycerate kinase | Y |
|  |  |  |  | Inclusion:21 |
|  |  |  |  | Exclusion:3 |

**Supplementary Table 1:** List of serine and glycine metabolism-related genes before and after exclusion.
