# Supplementary figures and images for: Serine and glycine metabolism-related gene expression signature stratifies immune profiles of brain gliomas, and predicts prognosis and responses to immunotherapy
Source: Front Pharmacol. 2022 Nov 17;13:1072253. doi: 10.3389/fphar.2022.1072253 (PMC9712738; doi:10.3389/fphar.2022.1072253)

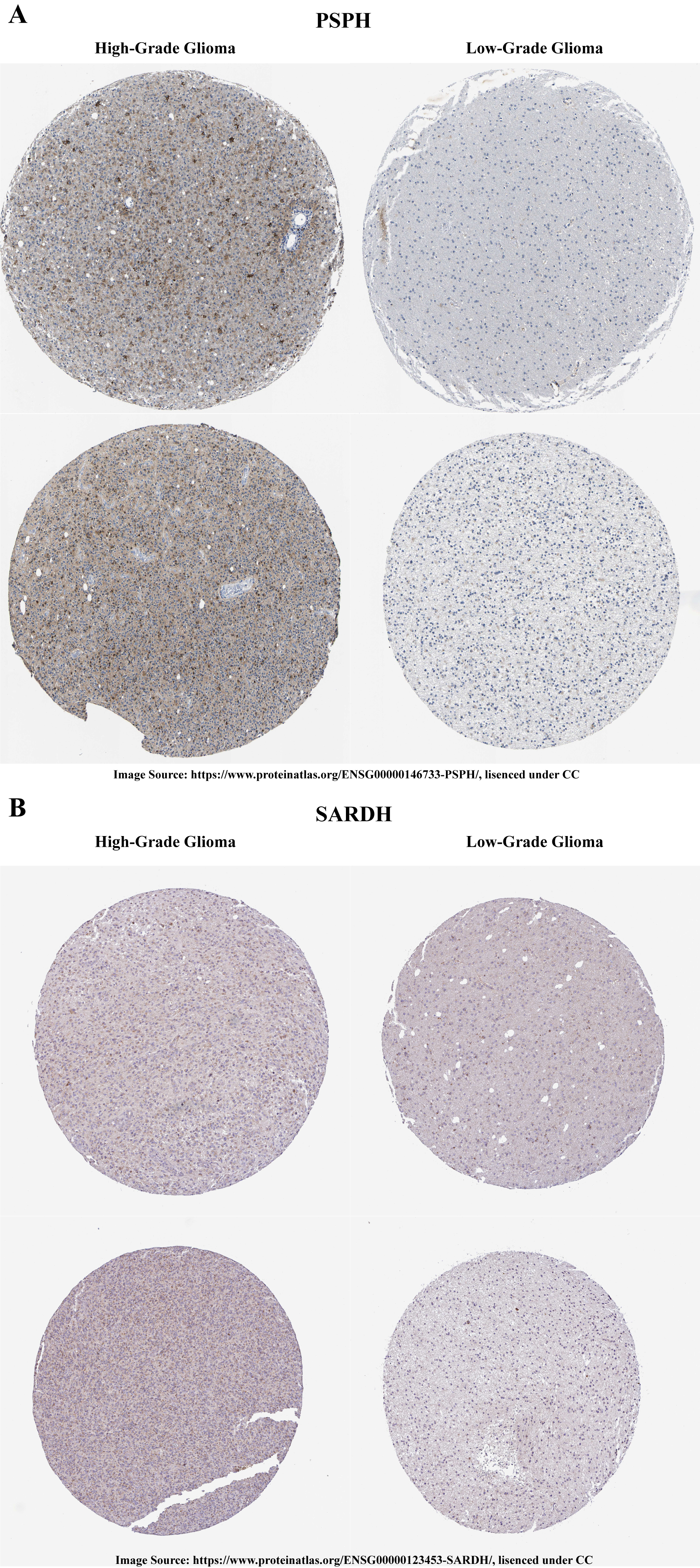

Supplement: Supplementary file 2 [file Image3.TIF]

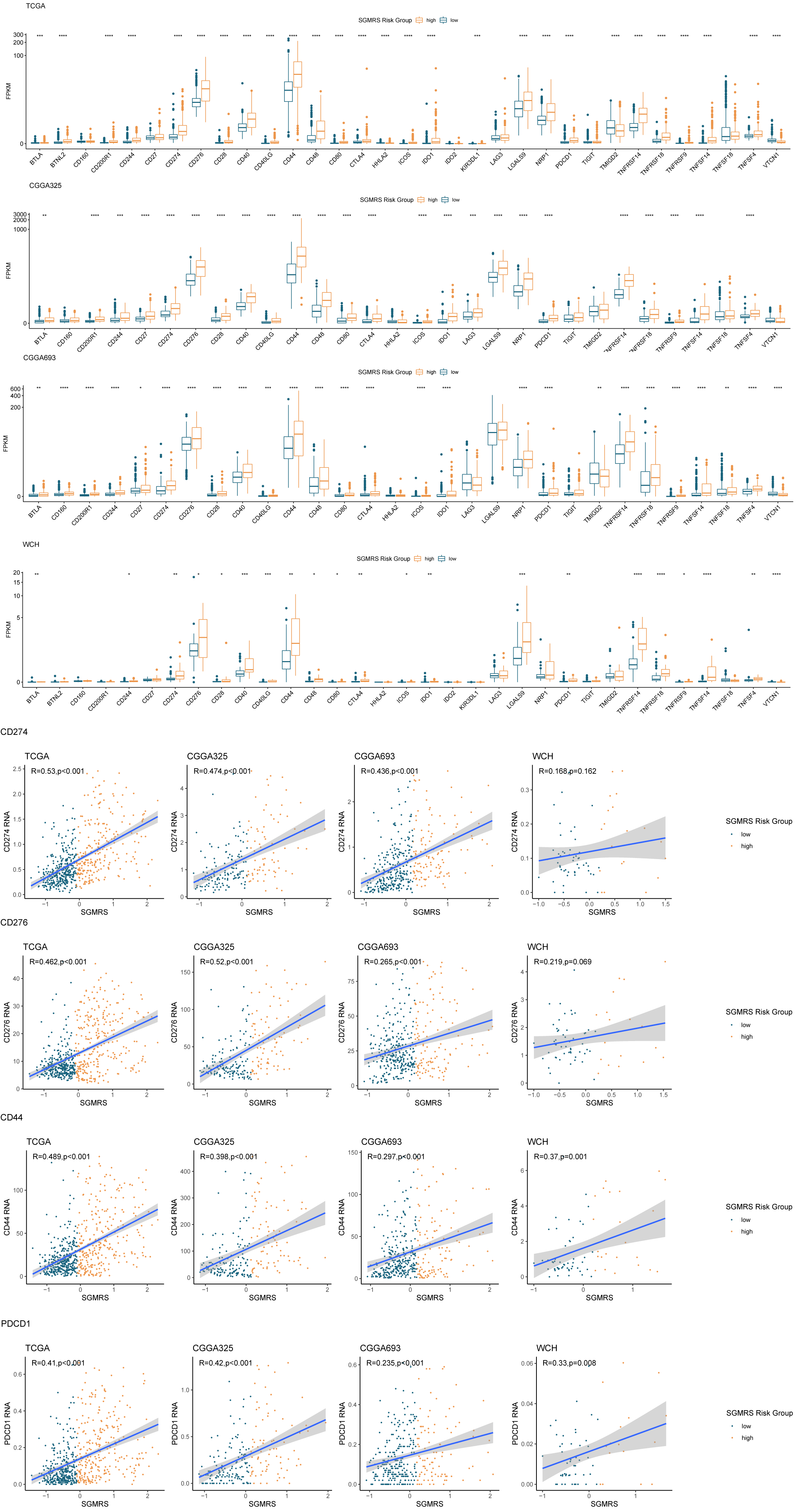

Supplement: Supplementary file 3 [file Image4.TIF]

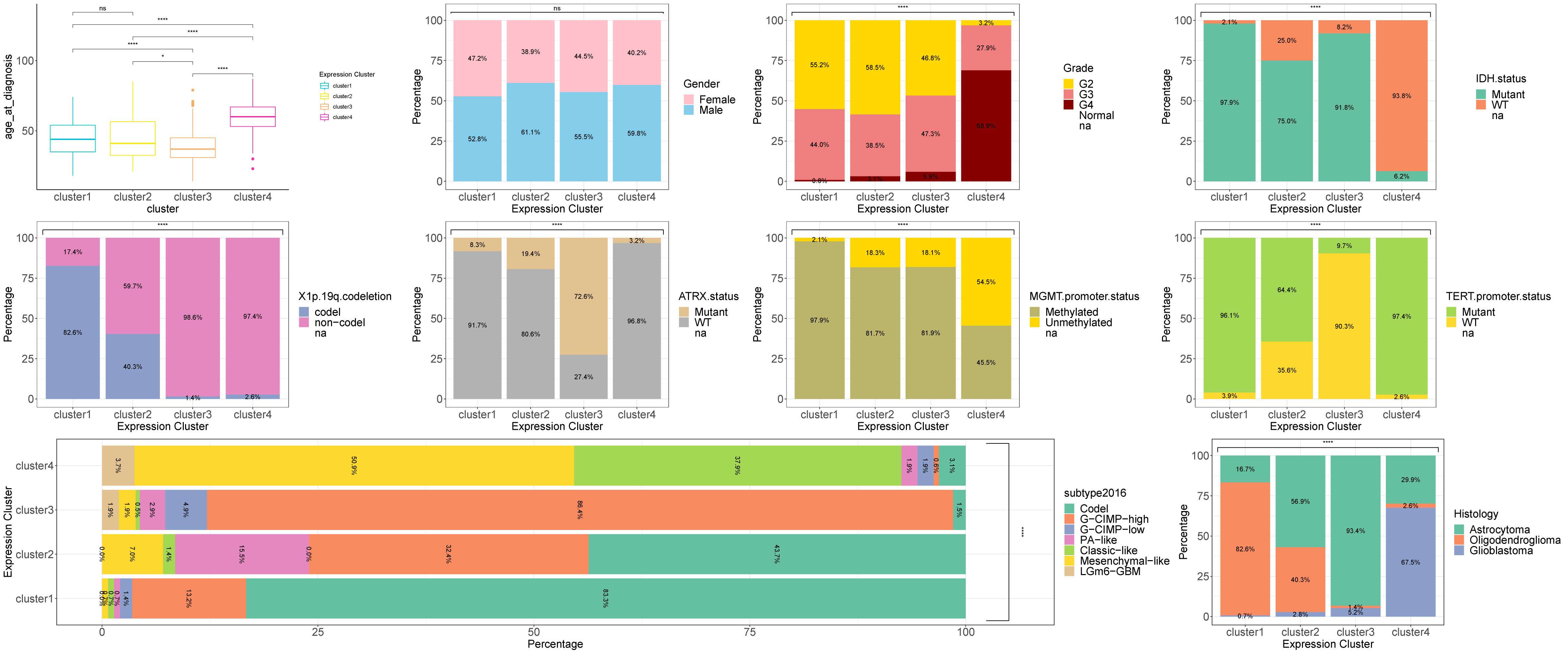

Supplement: Supplementary file 4 [file Image2.TIF]

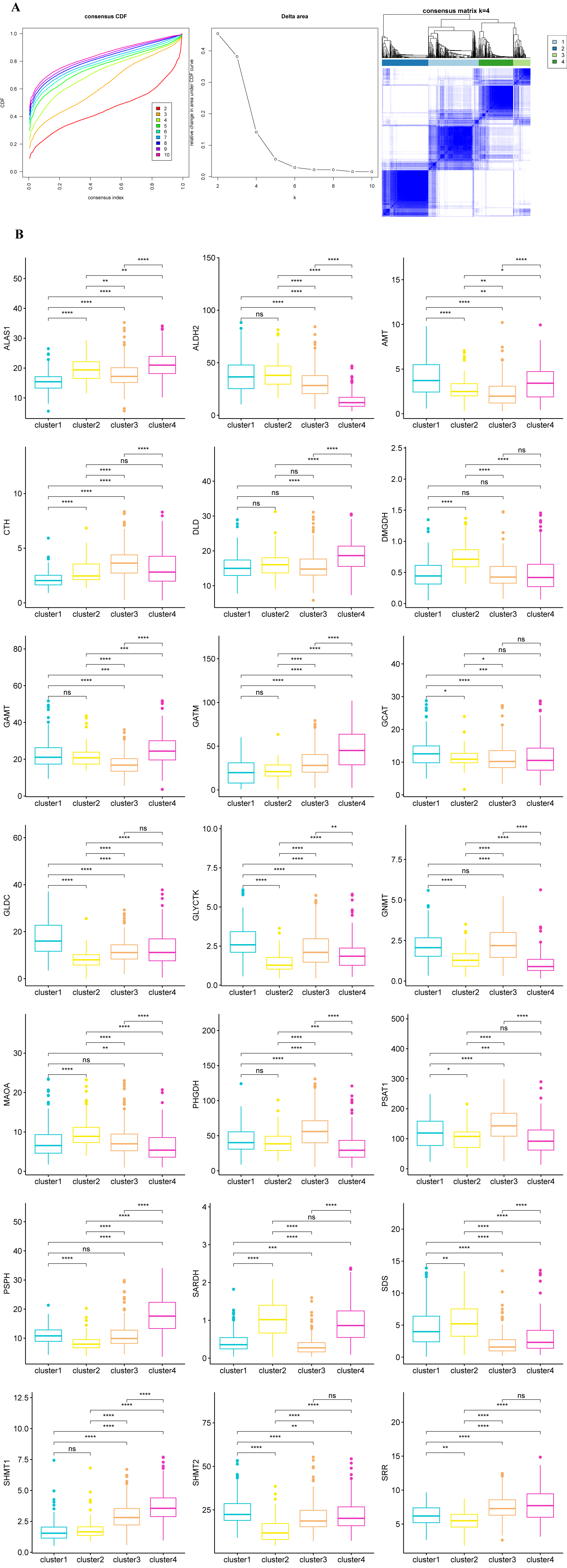

Supplement: Supplementary file 5 [file Image1.TIF]
